# Cough syrups: silent killer of Gambian children

**DOI:** 10.1097/JS9.0000000000000057

**Published:** 2023-02-16

**Authors:** Hitesh Chopra, Mohamed S. Attia, Syed Faisal Badshah, Kuldeep Dhama, Talha B. Emran

**Affiliations:** a Chitkara College of Pharmacy, Chitkara University, Rajpura, Punjab; b Department of Pharmaceutics, Faculty of Pharmacy, Zagazig University, Zagazig, Egypt; c Faculty of Pharmacy, The University of Lahore, Lahore, Pakistan; d Division of Pathology, ICAR-Indian Veterinary Research Institute, Bareilly, Izatnagar, Uttar Pradesh, India; e Department of Pharmacy, BGC Trust University Bangladesh; f Department of Pharmacy, Faculty of Allied Health Sciences, Daffodil International University, Dhaka, Bangladesh

Highlights

- Hundreds of child deaths from acute kidney damage were linked to the use of paracetamol syrup.
- Methylergometrine tablets were discovered to be counterfeit.
- Experts in the medical field have warned that due to the compounds’ solubility.
- Due to the widespread use of diethylene glycol, a poisoning epidemic broke out in the USA.

The WHO issued a health warning on October 5 over four items ‘that fail to fulfill either their quality criteria or specifications.’ This included Promethazine Oral Solution, Kofexmalin Baby Cough Syrup, Makoff Baby Cough Syrup, and Magrip N Cold Syrup, all of which had Maiden Pharmaceuticals Limited, Haryana, India, listed as their manufacturer^1^. The advisory continued by stating that the low-quality items indicated were dangerous and that their usage, especially by children, may ‘end in serious harm or death.’ As per the company’s website, the company also exports its medicines to countries in Southeast Asia, such as Laos, Vietnam, Thailand, Cambodia, the Philippines, Malaysia, and Indonesia, among others. It also has footprints in South American countries such as Ecuador, Chile, Venezuela, Suriname, and Paraguay, among others. It is also present in Russia, Poland, and Belarus^2^.

At the beginning of September, health officials in Gambia, a West African country, were investigating whether hundreds of child deaths from acute kidney damage were linked to the use of paracetamol syrup for fever, cough, cold, and discomfort. As of late July, doctors had seen an uptick in the frequency of instances of serious kidney injury in children younger than five, and they suspected a connection with drugs. Samples of all four goods were analyzed by the United Nations health agency, and the results showed that diethylene glycol (DEG) and ethylene glycol were present in ‘unacceptable quantities.’ These four syrups were believed to have been discovered in the Gambia, although it was possible that they had been unofficially disseminated to other markets. On September 29, the WHO notified the Indian regulator, the Drug Controller General of India, that it was advising and assisting the Gambia with this matter and offering technical help. The WHO warns that ingesting either DEG or ethylene glycol can be lethal^3^. The FDA (Food and Drug Administration) issued a warning detailing the potentially fatal consequences of the two substances, including discomfort, vomiting, diarrhea, difficulty passing urine, headache, a changed mental state, and severe renal damage (Fig. 1).

**Figure 1:**
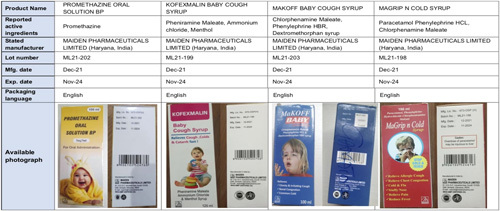
Details of products banned by WHO manufactured by Maiden Pharmaceuticals Limited. Image taken from Twitter.

Earlier in the year 2011, this company was blacklisted from Vietnam due to nongood manufacturing practices violations. According to the report, the Bihar government blacklisted Maiden Pharmaceuticals in 2011 for supplying substandard drugs. Methylergometrine tablets, which were taken from a hospital in Munger, were discovered to be counterfeit. Another batch of drugs, erythromycin sterate syrup 125 mg/5 mL, was found to be of unsatisfactory quality. According to the extended licensing, laboratory, and legal node (XLN) database maintained by the Government of India, the Kerala, and Gujarat state governments have repeatedly issued warnings against the company’s illegal practices. A Kerala drug inspector even filed a case in 2005, after which the company was fined in 2017. In another case, a central government drug inspector prosecuted the company in Sonipat for quality violations under the Drugs and Cosmetics Act. The company was charged with offenses related to adulteration.

This was the fourth case of mass glycol poisoning in India. In 1973, there was a similar incident at the Children’s Hospital, Egmore, in Chennai that caused the deaths of 14 children. A similar poisoning at Mumbai’s J.J. Hospital in 1986 killed 14 patients who were otherwise on the mend. In 1998, 33 children died in two hospitals located in New Delhi due to similar poisoning.

Experts in the medical field have warned that due to the compounds’ solubility, DEG and ethylene glycol may be accidentally contaminated when they are substituted by less expensive commercial-grade versions of the same solvents that also include DEG and ethylene glycol.

Solvents such as DEG and ethylene glycol, both of which are banned in the USA, are sometimes used to illegally adulterate liquid medications. The United States National Center for Biotechnology Information reports that glycerin (also known as glycerol) and propylene glycol are frequently used as solvents in cough syrups because they provide a liquid base for the nonwater-soluble paracetamol or acetaminophen.

Due to the widespread use of DEG as a solvent, a poisoning epidemic broke out in the United States in 1937. Many children were among the 107 fatalities. As a result of this, Congress passed the Federal Food, Drug, and Cosmetic Act (the Act), which includes a clause demanding proof of a drug’s safety before it may be sold. Hospitals in Port-au-Prince, Haiti, saw an influx of young people in late 1995 and early 1996 due to cases of acute renal failure; at least 80 of them died. Glycerin in acetaminophen syrup made in Haiti was tainted with DEG, according to an investigation by Haitian health officials, the CDC (Centers for Disease Control and Prevention), and the FDA. Hundreds of children died after DEG poisoning incidents between 1990 and 1998 in Argentina, Bangladesh, India, and Nigeria. A poisoning epidemic caused by DEGs occurred in Panama in October 2006, causing several illnesses and deaths. Due to the emergence of this type of case, the FDA issued guidance in 2007 for the testing of glycerin.

Learning from this kind of case, the Indonesian Government, on October 19, 2022, laid a temporary ban on all liquid medicines in the country over a reported spike in children’s deaths linked to medical syrups. On October 18th, news came out from Indonesia that 99 out of 206 young people with acute renal failure in 20 different regions had passed away. As the health ministry continues its investigations into unlicensed medicinal syrups supplied in the country, that number is projected to grow further, having already reached 133.

Apart from this sequence, the major question is why this happened. What led to this incident? When the product is released in the market, it is accompanied by a certificate of analysis that shows the tests done on the product as per the specifications or pharmacopeia to which it has been subjected. That certificate is signed by an FDA-approved person (a quality control chemist) only. And for the registration of a product in a certain country, a dossier needed to be sent mentioning all the sources of raw material and packing material and their roles in formulation. Apart from this, the dossier contains another necessary document. These documents are sent to regulatory agencies for product approval.

Now discussing these documents, which were missed during the approval and export of the product, If the regulatory agency had gone through these documents properly, these kinds of things could have been avoided. Based on documents, the company supplying the raw material containing the DEG should also be investigated; may be they have a higher content of DEG in their product itself.

As a precaution, the regulatory agencies should go for audit checks regularly and ask the manufacturers for proper documentation. Also, the regulatory agencies of the importing country should establish satellite testing laboratories for testing the product from each consignment getting shipped. Though it will increase the time taken to get the product exported, surely it will save lives.

## Ethical approval

Not applicable.

## Sources of funding

None.

## Author contributions

H.C.: conceptualization, data curation, writing – original draft preparation, writing – reviewing and editing. M.S.A., S.F.B., K.D.: data curation, writing – original draft preparation, writing – reviewing and editing. T.B.E.: writing – reviewing and editing, visualization, supervision.

## Conflicts of interest disclosure

The authors declare that they have no financial conflict of interest with regard to the content of this report.

## Research registration unique identifying number (UIN)

None.

## Guarantor

Talha Bin Emran, PhD, Associate Professor, Department of Pharmacy, BGC Trust University Bangladesh, Chittagong 4381, Bangladesh. Tel: +880 303 356 193, Fax: +880 312 550 224. https://orcid.org/0000-0003-3188-2272 and Department of Pharmacy, Faculty of Allied Health Sciences, Daffodil International University, Dhaka 1207, Bangladesh. E-mail: talhabmb@bgctub.ac.bd

## Provenance and peer review

Not commissioned, internally peer-reviewed.

## Data statement

No specific data collected for the above manuscript. The data in this correspondence article is not sensitive in nature and is accessible in the public domain. The data is therefore available and not of a confidential nature.
